# Muscle Biopsy: A Requirement for Precision Medicine in Adult-Onset Myopathy

**DOI:** 10.3390/jcm11061580

**Published:** 2022-03-13

**Authors:** Meng-Ju Wu, Wei-An Liao, Po-Yu Lin, Yuan-Ting Sun

**Affiliations:** 1 Department of Neurology, National Cheng Kung University Hospital, College of Medicine, National Cheng Kung University, Tainan 704, Taiwan; topmoslover@gmail.com (M.-J.W.); pylin1991@gmail.com (P.-Y.L.); 2 Department of Pathology, National Cheng Kung University Hospital, College of Medicine, National Cheng Kung University, Tainan 704, Taiwan; i5493133@gmail.com; 3 Department of Medical Genomics, National Cheng Kung University Hospital, College of Medicine, National Cheng Kung University, Tainan 704, Taiwan

**Keywords:** muscle biopsy, idiopathic inflammatory myopathies, muscle pathology, precision medicine, adult-onset myopathy

## Abstract

Muscle biopsy is a fundamental procedure to assist the final diagnosis of myopathy. With the recent advances in molecular diagnosis, serology tests, and mechanism-based classification in myopathy, the précised diagnosis for myopathy required the applications of multiple tools. This study intends to reappraise the benefit of muscle biopsy in adult-onset myopathy under the setting of an optimized muscle biopsy protocol and comprehensive serology tests. A one-group pretest-posttest study design was used. The pre- and post-biopsy diagnoses and treatments in 69 adult patients were compared. Muscle biopsy yielded 85.5% of definitive diagnoses, including changes in pre-biopsy diagnoses (40.6%) and narrowing down the suspicious myopathies (49.3%). The demographic data and clinical parameters between the group “with change” and “without change” after biopsy were not different. Among those with changes in diagnosis, 39.3% also had a corresponding shift in treatment, which benefits the patients significantly. Regarding the most common adult-onset myopathy, idiopathic inflammatory myopathy (IIM), 41% of patients with pre-biopsy diagnosis as IIM had changes in their IIM subtype diagnosis, and 53% was finally not IIM after muscle biopsy. Although there have been advances in molecular diagnosis recently, muscle biopsy still undoubtedly critically guided the diagnosis and treatment of adult-onset myopathy in the era of precision medicine.

## 1. Introduction

Muscle biopsy is a fundamental procedure to assist the final diagnosis of myopathy. Recently, there have been considerable advances in the diagnosis and treatments for adult-onset myopathy. For instance, the newly identified autoantibodies for serology and molecular markers for muscle histopathology [1,2,3,4] prompted the change of classifications for idiopathic inflammatory myopathy (IIM) [5,6]. Achievements in high throughput genomic technologies, such as whole exome sequence, in conjunction with the advances in molecular studies, have increased the diagnostic yield for hereditary myopathies [7,8,9,10,11] and facilitated the re-classification of limb-girdle muscle dystrophy [12,13,14,15,16]. Although the aforementioned improvements brought about a more precise pre-biopsy diagnosis of myopathy, the essential role of muscle biopsy in the final diagnosis may still not be substituted [17,18,19,20,21].

**Figure 1:**
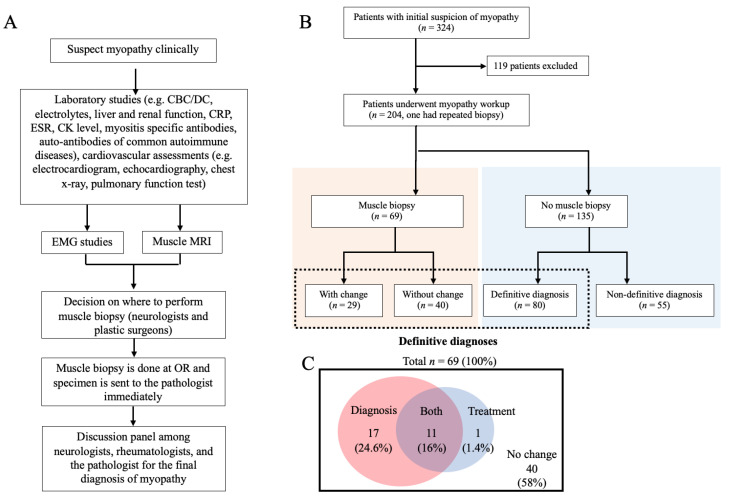
(**A**) A flowchart of the standardized diagnostic workup for clinically suspected myopathy at National Cheng Kung University Hospital. CBC, complete blood count; DC, differential count; CRP, C-reactive protein; ESR, erythrocyte sedimentation rate; CK, creatine kinase; EMG, electromyography; MRI, magnetic resonance imaging; OR, operating room. (**B**) A flowchart for patients with initial suspicion of myopathy between 1 January 2018 and 25 October 2021. One patient had repeated biopsy, 119 patients who did not have final diagnosis of myopathy or <18 years old were excluded. Finally, 204 patients who underwent comprehensive diagnostic workup for myopathy were classified into two groups according to muscle biopsy. The orange rectangle represented patients underwent muscle biopsy. The blue rectangle represented patients who did not have muscle biopsy. 1 patient was excluded from the biopsy group because of repeated biopsy. A total number of 69 patients were further classified into the group with change and the group without change. The patients who had a final definitive diagnosis were included in the rectangle with dotted lines. *n*, number of patients; EMR, electronic medical record. (**C**) A Venn diagram for the distribution of patients in change of treatment, change of diagnosis, and change of both diagnosis and treatment. Numbers represent the numbers of the patients involved.

This study intends to reappraise the benefit of muscle biopsy in adult-onset myopathy under the setting of an optimized muscle biopsy protocol and comprehensive serology tests (Figure 1A). The objective was to evaluate the effect of the invasive procedure, the muscle biopsy, in altering the final diagnosis and treatment of patients who were previously diagnosed with certain types of myopathies. The results may assist neurologists and rheumatologists in conducting shared decision-making with patients with myopathy.

## 2. Method

### 2.1. Study Design

This is a retrospective observational case-control study nested with a one-group pretest-posttest design, particularly for the muscle biopsy group. The study protocol was approved by the Institutional Review Board (IRB) at National Cheng Kung University Hospital (NCKUH) (A-ER-110-453). Owing to the retrospective nature of the study, the IRB waived the requirement for informed patient consent. The effects of intervention, muscle biopsy, were evaluated by the ratio of changes in diagnosis and treatments between pre- and post-biopsy. The two parameters, change in diagnosis and treatment, were neither mutually exclusive nor simultaneously present entirely—most of the time, the evolution of diagnosis results in a shift in treatments. However, sometimes only treatment plans were changed by muscle pathology.

### 2.2. Patients

Adult patients who underwent their first muscle biopsy due to clinical suspicion of myopathy at the NCKUH between 1 January 2018 and 25 October 2021, were enrolled. Patients who had clinical suspicion of myopathy but did not undergo muscle biopsy were identified through electronic medical records, and they were assigned to the control group. Relevant demographic information, creatine kinase (CK) level documented on the pathology report or at the initial clinical encounter, main finding(s) from electromyography (EMG) study, findings of muscle magnetic resonance imaging (MRI), pre-biopsy clinical diagnosis and treatment, and post-biopsy clinical diagnosis and treatment were carefully collected.

### 2.3. Muscle Biopsy Procedure and Interpretation

#### 2.3.1. Muscle Biopsy Procedure

After confirming myopathy by EMG and nerve conduction studies (NCS), the neurologist and the plastic surgeon decided the most appropriate location for muscle biopsy with the assistance of muscle MRI, which was usually the thigh muscles. The open muscle biopsy was carried out in an operating room, and the patients underwent local anesthesia. As soon as the muscle specimen was obtained, it was processed as follows: the sample was placed on the cork base, fixed with OCT mounting medium (Merck), freezing with liquid nitrogen, cooled for solidity, and then cut into sections [22]. The processed sample was read by a qualified pathologist specializing in neuromuscular diseases. The descriptions of each specimen included the gross findings, the microscopic finding, and the ultrastructural findings. In addition to hematoxylin and eosin (H & E) stain, frozen sections with special stains were applied to each sample, including Gomori Trichrome, ATPase (4.3, 4.6, and 9.4), nicotinamide adenine dinucleotide dehydrogenase-tetrazolium reductase (NADH-TR), Sudan III, oil-red-O, periodic acid-Schiff (PAS), Cytochrome c oxidase (COX) and succinate dehydrogenase (SDH). Staining with Congo red, beta-amyloid, and acid-fast stains were performed on selected cases.

#### 2.3.2. Histopathology Diagnosis

All slices were viewed by a qualified muscle pathologist according to Chunyu Cai et al. (2019) [23]. The diagnosis of inflammatory myopathy required the presence of active myopathic damage and inflammation. Active myopathic damages include the presence of rounded atrophic fibers, random change of fiber size, fiber necrosis, and phagocytosis. The “inflammation” stands for lymphocytic infiltrate. Immunostains including CD3, CD4, CD8, CD20, and CD68 were used to identify the types of inflammatory cells. Pan-T cells are labeled by CD3. T helper cells are labeled by CD4. Granulocytes and macrophages may show weak positivity of CD4. Cytotoxic T cells are labeled by CD8. Mature B cells are labeled by CD20. Macrophages are labeled by CD68 [24]. In patients who received immune therapy, the lymphocytic infiltration can be scarce or restricted to the perivascular area, and the active myopathic damage can be limited. Thus, we used immunostain of major histocompatibility complex (MHC) class I to support autoimmune pathogenesis. On the other hand, if the pathology reveals myofiber necrosis without or with minimal infiltration of inflammatory cells, an alternative diagnosis such as immune-mediated necrotizing myopathy (IMNM) would be considered. Metabolic myopathies were recognized by “myopathies with distinctive inclusions or vacuoles.” Cytoplasmic lipid droplets were displayed by Sudan III and oil-red-O stains. Glycogen deposition was identified by PAS stain. Staining for p62 was introduced for clinically suspected inclusion body myositis (IBM). An electron microscope for visualizing ultrastructural-abnormality was used for diagnosing vacuolar myopathy or hereditary myopathies. While excessive cytoplasmic lipid droplets presented, suggesting lipid-related disorders and mitochondrial myopathies [22,25], fatty acid enzyme analysis or genetic studies was introduced for further clarification. A negative muscle biopsy result does not entirely exclude metabolic myopathy, since in some cases, such as carnitine palmitoyltransferase II deficiency, histology can appear normal while no recent flare-up [23]. A comprehensive discussion of the final diagnosis was made on a monthly held expert meeting in which neurologists, pulmonarists, rheumatologists, and pathologists are attended (Figure 1A).

### 2.4. Clinical Information and Diagnostic Criteria

#### 2.4.1. NCS and EMG

Certified technicians performed NCS before every EMG study. The EMG was conducted over four limbs by practicing neurologists at NCKUH according to Paganoni et al. (2013) [26]. In the upper limb, one proximal muscle (e.g., deltoid or biceps brachii) and one distal muscle (e.g., first dorsal interosseous or abductor pollicis brevis) were included. In the lower limb, one proximal muscle (e.g., rectus femoris, vastus medialis, or other) and one distal muscle (e.g., tibialis anterior or gastrocnemius) were included. Muscles innervated by NCS-shown diseased nerves were avoided to minimize misinterpretation. The descriptions of EMG findings included the type and quantity of rest potential, the amplitude and duration of motor unit action potential, the interference pattern, and the presence of early recruitment. The crude classification of normal, myopathy, neuropathy, or mixed type was based on the aforementioned parameters. The mixed type indicated the presence of both neuropathic and myopathic changes.

#### 2.4.2. MRI

Muscle MRI routinely used for myopathy patients helped recognize the specific involvement patterns in certain hereditary myopathy [27,28,29] and guided the location for a muscle biopsy to minimize the sampling bias [30]. The MRI protocol comprised four sequences: (a) axial view of T1-weighted image (T1WI) with fast spin-echo (FSE), (b) axial view of proton-density-weighted image (PDWI) with fat suppression and FSE, (c) coronal view of T2-weighted image (T2WI) with fat suppression and FSE, and (d) sagittal, axial, and coronal views, contrast-enhanced, T1WI with fat suppression and FSE. The T1WI identifies fatty infiltration location, and the T2WI discloses fascia in active damaging by high water content [31]. The descriptions of MRI included the symmetry of muscle bulk, the distribution of abnormal signals, such as edema, fatty replacements, and increased contrast enhancement in lower limbs, the configurations and the signal intensity of the facias, bones, the joints, the bone marrow, and the soft tissue. Lower limbs muscle MRI instead of whole-body MRI was most often used in all our subjects because thigh muscles are most commonly affected in IIMs (66–86% sensitivity) [32,33,34,35], and the time-cost is efficient [36]. The probability of inadequate sampling for biopsy was minimized with the standardized imaging and EMG protocols. All muscle MRI images were interpreted by two radiologists specializing in musculoskeletal systems.

#### 2.4.3. Diagnosis of IIMs

IIMs and the subgroups were defined according to 2017 EULAR/ACR classification criteria [20]. Anti-synthetase syndrome (ASS) was defined by Solomon et al. (2011) with the presence of anti-aminoacyl tRNA synthetase antibody plus two major or one major with two minor criteria [37]. Overlap myositis or overlap syndrome (OM) was defined by Troyanov et al. (2005) with the presence of myositis in addition to the connective tissue disease [38]. Autoimmune Inflammatory Myopathies 16 Ag (IgG) immunoblot kit (EUROIMMUN), which included the tests for antibodies against Mi-2α, Mi-2β, TIF1γ, MDA5, NXP2, SAE1, Ku, PM-Scl 100, PM-Scl 75, Jo-1, SRP, PL-7, PL-12, EJ, OJ, and Ro-52, was used in serology test for patients suspected with IIM.

#### 2.4.4. Genetic Tests

Genetic tests for patients with myopathy included whole-exome sequencing (WES) for point mutation, mass spectrometry, and Southern blot for repeats. WES and the following analysis were conducted with a Next-Generation Sequencing platform consisting of Illumina NovaSeq 6000 (Illumina, Inc. San Diego, CA, USA) for librarying, DRAGEN 3.7.5 (Illumina, Inc.) for variant calling, Ensembl Variant Effect Predictor (version 100), and Jan-novar (version 0.35) with dbNSFP 4.1a for annotation. The Human Phenotype Ontology (HPO) terms including “myopathy” or “muscle” were adopted to rank the disease-associated genes.

#### 2.4.5. Other Criteria

Toxin or drug-related myopathy was the occurrence of myopathy secondary to any myotoxic agent by Dalakas (2009) [39]. Sarcopenia was defined according to the 2019 Asian Working Group for Sarcopenia (AWGS) [40]. The typical pathology was the extensive reduction in type II muscle fiber size and number [41,42] without inflammatory cells or vacuoles.

All myopathies were classified into four main categories of diagnosis: inflammatory/autoimmune, toxin/endocrine, metabolic/mitochondrial diseases (M/M), and others. Muscular dystrophies and congenital myopathies were assigned into the category of “others,” in considering the rarity, with the prevalence of 16.14/100,000 and 1/25,000, respectively, and childhood-onset nature [43,44,45]. When there were several differential diagnoses before the biopsy, the most likely diagnosis was chosen. The diagnosis was considered “changed” while the main category or the subtype of a specific category was changed after muscle biopsy. There are four main categories of the treatment: steroid and/or immune-modulating therapy, discontinue the myotoxic agent, other types of medication, and no treatment. The treatment was considered “changed” while the primary treatment or the dosage of the existing therapy was changed, or additional medication was provided.

### 2.5. Statistical Analysis

Statistical analyses were performed using Prism (version 6; GraphPad Software, La Jolla, CA, USA). Unpaired Student’s *t*-test, Mann–Whitney U test, or Fisher’s exact test were used according to data type. Normality tests were conducted for continuous data before comparisons. Significance was set at *p* < 0.05.

## 3. Results

From 1 January 2018 to 25 October 2021, there were 324 patients with suspicion of myopathy. After excluding the minors, a patient who had repeated biopsy, and those identified as non-myopathy diagnoses, medical records of 204 subjects who underwent myopathy workup were reviewed. Among them, 69 patients were included for the pretest-posttest analysis (Figure 1B). In patients with biopsy, male patients accounted for 39%, and the average age was 54 ± 13.7 years old. The average CK level was 3031 U/L (ranged 14–55,716 U/L). In patients without biopsy, male patients accounted for 43.7%, and the average age was 49.8 ± 15 years old.

Muscle biopsy changed the diagnosis and treatment in 42% and 33% of myopathy patients, respectively. We assigned 29 patients into “with change” group who had either changes in diagnosis (*n* = 25) or treatment (*n* =12) after biopsy (Figure 1B). Among them, 11 had changes in both diagnosis and treatments (Figure 1C). Patients allocated into “without change” group (*n* = 40, 58%) had neither change in diagnosis nor in treatment after biopsy.

The demographic data (age, gender) and clinical data (CK levels) between the group “with change” and the group “without change,” were not different (age, gender, and CK levels, *p* = 0.258, *p* = 0.126, and *p* = 0.495, respectively, Table 1). The ratio of positive myopathic changes in EMG was equal (62.3% in the entire cohort, 62.5% in the unchanged group, and 62.1% in the changed group, *p* = 0.517, chi-square test, Table 1). The abnormal signals disclosed on muscle MRI was not different (66.7% of the entire cohort, 62.5% of the unchanged group, and 72.4% of the changed group, *p* = 0.909, chi-square test, Table 1). Before muscle biopsy, the most common diagnosis was IIM, which accounted for 62% of adult patients with myopathy. Steroid or immune-modulating therapy was also the most common treatment before muscle biopsy (55.1% in the entire cohort, 60% in the unchanged group, and 48.3% in the changed group, Table 1). Eighteen patients (26%) did not receive any treatment before the biopsy. The distribution of pre-biopsy diagnosis and pre-biopsy treatment were equal between the changed and the unchanged groups (*p* = 1.000 and *p* = 0.990, respectively, Chi-square test, Table 1). In patients with a change of diagnosis, 17 (60%) did not have an accompanying shift in treatment, 11 had changes in both the diagnosis and treatment (39.2%). There was only one patient (3%) whose treatment was changed without diagnosis change. Then we analyzed how the diagnosis and treatments changed by muscle pathology as follows.

### 3.1. The Changes of Pre- and Post-Biopsy Diagnoses

**Figure 2:**
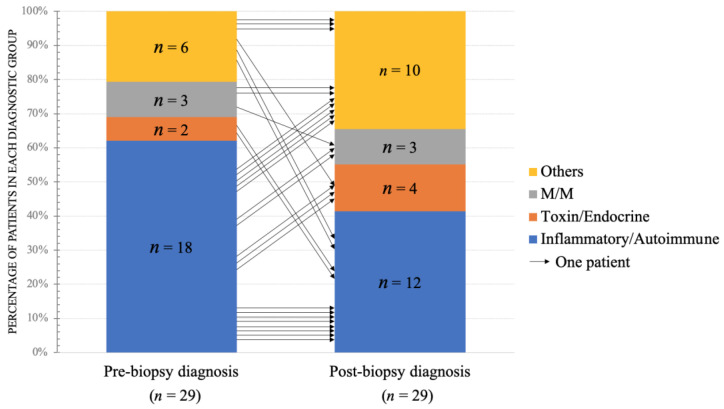
Comparison of pre- and post-biopsy clinical diagnoses in the group with change. A total number of 29 patients in the group with change was demonstrated. The vertical axis represented the percentage of patients in each diagnostic group. Each arrow represented one patient and showed the corresponding change of diagnosis after muscle biopsy. *n*, number of patients. M/M, metabolic/mitochondrial diseases.

The evolutions of pre- and post-biopsy diagnoses are shown in Figure 2. The direction of arrows was from pre-biopsy diagnosis to post-biopsy diagnosis. The pre-biopsy distribution of inflammatory/autoimmune myopathies, toxin/endocrine myopathies, M/M, and others was 62% (18 of 29), 7% (2 of 29), 10% (3 of 29), and 21% (6 of 29), respectively. After the biopsy, the distribution of the aforementioned four categories was changed to 41% (12 of 29), 14% (4 of 29), 10% (3 of 29), and 35% (10 of 29), respectively. The patient number of IIM was reduced while that of the other three categories of myopathy was increased.

IIM was the most common myopathy diagnosis in adults, whether before or after a biopsy. There were finally 12 patients who got the definite diagnosis of IIM. Among them, four obtained the diagnosis by pathology. One with severe rhabdomyolysis of the unspecified cause was confirmed as an IMNM, another with pre-existing interstitial lung disease (ILD) suspected of having critical illness polyneuropathy was finally confirmed as ASS. One was initially thought hyperthyroidism-related weakness and finally proved to be myositis. One had pre-existing myositis with long-lasting use of steroids whose diagnosis was changed from steroid-myopathy to a flare-up of myositis.

On the other hand, nine that were misdiagnosed as IIMs before muscle pathology were reclassified into different categories according to muscle pathology: two were steroid-related myopathy, two were M/M myopathy, one was sarcopenia, one was mycobacterium-related granulomatous myopathy, one was muscular dystrophy, and two were not myopathy (fibromyalgia, diabetic amyotrophy). The details of the aforementioned misdiagnosed cases are summarized in Table 2. To analyze factors interfering with the accuracy of diagnosis, in addition to the unavoidable IIM mimicking presentations, 60% (*n* = 6) patients had weak to strong positivity of myositis-associated antibodies. Some might be related to patients underlying diseases; some might be derived from the false positivity of the serology kit.

IIM, idiopathic inflammatory myopathy; NE, neurological examination; T2DM, type 2 diabetes mellitus; MRC, Medical Research Council scale for muscle strength; CK, creatine kinase; NCS, nerve conduction study; EMG, electromyography; NE, neurological examination; MRI, magnetic resonance imaging; MADD, multiple acyl-CoA dehydrogenation deficiency; MS/MS, mass spectrometry; SRP, signal recognition particle antibody; SAE1, small ubiquitin-like modifier activating enzyme; ILD, interstitial lung disease; MHC, major histocompatibility complex; COX, Cytochrome *c* oxidase; SDH, succinate dehydrogenase; AMPDA, adenosine monophosphate deaminase; NADH-TR, nicotinamide adenine dinucleotide dehydrogenase-tetrazolium reductase.

### 3.2. The Changes of Pre- and Post-Biopsy Treatments

**Figure 3:**
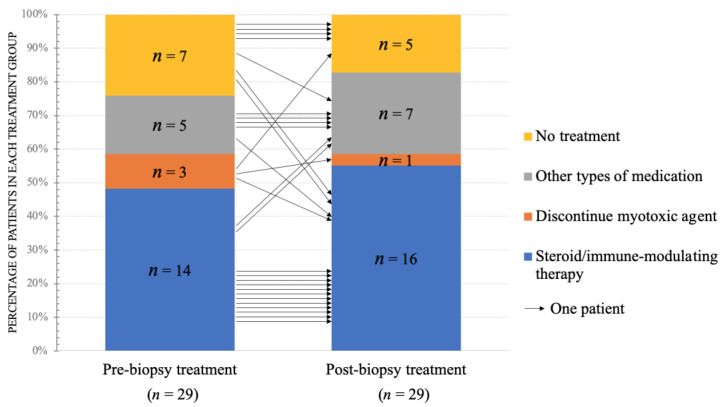
Comparison of pre- and post-biopsy clinical treatment in the group with change. A total number of 29 patients in the group with change was demonstrated. The vertical axis represented the percentage of patients in each treatment group. Each arrow represented one patient and showed the corresponding change of treatment after muscle biopsy. *n*, number of patients.

The evolutions of pre- and post-biopsy treatment are shown in Figure 3. Arrows indicated the flow of patients from pre-biopsy to post-biopsy treatment. Before muscle biopsy, 48% (14 of 29) of patients were undergoing steroid/immune-modulating therapy, 10% (3 of 29) were advised to discontinue myotoxic agents, 17% (5 of 29) were with other treatments, and 24% (7 of 29) were without any treatment. After muscle biopsy, the patient number in the “no treatment” group was reduced. More patients received steroid/immune-modulating therapy (55%) and other types of treatments (24%). In total, 11 patients had a change in therapy after biopsy, 8 were changes in the category of treatment, 3 were changes in the dosage of the original medicine. The detailed clinical information of the 11 patients mentioned above was summarized in Table 3.

The reasons for changing therapy in these patients included: (1) The diagnosis was changed. For instance, the steroid was replaced with carnitine due to the final diagnosis of multiple acyl-CoA dehydrogenation deficiency (MADD) in one patient and replaced with anti-mycobacterial treatment in another patient who was confirmed as mycobacterium-related granulomatous myopathy. (2) The change of disease activity. For instance, the immune-modulating therapy of three IIM patients was escalated after biopsy due to the progression of clinical signs and active inflammation found on histopathology. Regarding those without changing treatments, muscle pathology increased physicians’ confidence in caring for those patients, such as discontinuing myotoxic agents in patients with myotoxic myopathy and improving glycemic control in patients with diabetic amyotrophy.

### 3.3. The Special Considerations in IIM

**Figure 4:**
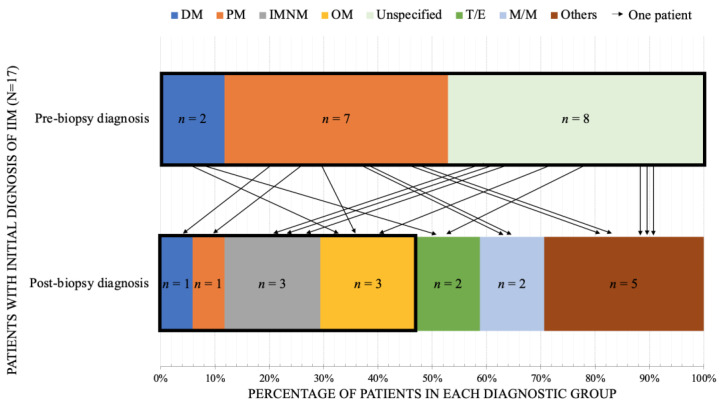
Change of subtypes or different types of diagnosis from pre-biopsy inflammatory myopathy. A total number of 17 patients with pre-biopsy diagnosis of IIM was demonstrated. The horizontal axis represented the percentage of patients in each diagnostic group. The thick border represented the collection of IIMs. Each arrow represented one patient and showed the corresponding change of diagnosis after muscle biopsy. The diagnosis of IIM was reduced by 53% after muscle biopsy was performed. More specific subgroups of IIMs were identified after muscle biopsy. IIM, idiopathic inflammatory myopathy; DM, dermatomyositis; PM, polymyositis; IMNM, immune-mediated necrotizing myopathy; OM, overlap myositis; M/M, metabolic/mitochondrial diseases; T/E, toxin/endocrine; *n*, number of patients.

The most prevalent adult-onset myopathy, the IIM, had recent advances in the subtype classification [17,46]. Thus, we analyzed how muscle biopsy affects the precise diagnosis of IIM subtypes, particularly in clinically indistinguishable cases. Arrows in Figure 4 indicate the flow of patients from pre-biopsy to post-biopsy diagnosis of IIM subgroup and other three diagnostic groups. In 17 clinical IIM patients, only one remained in the same subtype after biopsy. Muscle pathology provided an effect of precision subtyping in 94.1% of IIM patients. The pre-biopsy diagnoses of the other 16 patients were dermatomyositis (DM, *n* = 2), polymyositis (PM, *n* = 6), and unspecified subtypes (*n* = 8). After the biopsy, two DM patients were diagnosed with OM and steroid-related myopathy. Six patients with PM were diagnosed as DM, one as OM, two as M/M, one as muscular dystrophy, and one as sarcopenia. In IIM with unspecified subtypes, three patients were finally diagnosed as IMNM, one was OM, one was steroid-related myopathy, one was diabetic amyotrophy, one was mycobacterium-related granulomatous myopathy, and one was neuropathic change. To sum up, among patients with pre-biopsy diagnosis of IIMs, there was a 71% chance to be non-IIMs after muscle biopsy (odds ratio = 0.71, 95% confidence interval = (0.15, 3.33)). In addition, 53% of patients who shared indistinguishable features with IIMs obtained a more specific subtype diagnosis after muscle biopsy (Table 4).

### 3.4. Myopathy Patients without Muscle Biopsy

Since not all patients with a clinical impression of myopathy underwent muscle biopsy, we analyzed how the final diagnosis was made without muscle pathology in these myopathy patients. During the same period, 135 patients underwent comprehensive workup for myopathy except for muscle biopsy. Among them, 80 patients received a definitive diagnosis finally while 55 patients did not. Overall, the biopsy rate for patients with definitive diagnosis (*n* = 149) was 33.8%. The biopsy rate in each diagnostic group is as the followings: 53% (44/83) in IIM/autoimmune, 6.5% (2/31) in toxin/endocrine, 80% (8/10) in M/M, and 60% (15/25) in others.

Among the 80 patients with a definite diagnosis, the definitive diagnosis was confirmed by genetic tests (*n* = 12), presence of myositis-specific antibodies (*n* = 22), abnormal thyroid and/or cortisol level with treatment improvement (*n* = 22), clinical improvement with myositis treatment (*n* = 12), clinical improvement with discontinuing myotoxic agents (*n* = 7), diagnostic criteria of OM (*n* = 4), and skin biopsy of DM (*n* = 1). The distribution of the four main diagnosis categories is as the followings: 39/80 (48.7%) were IIM/autoimmune, 29/80 (36.3%) were toxin/endocrine, 2/80 (2.5%) were M/M, and 10/80 (12.5%) were in others group. The distribution was different from those who received a biopsy (listed in Table 1) (*p* < 0.0011, chi-square test). The difference indicated various weights of muscle pathology in different categories of muscle disease. For instance, diagnosis can be easily made by observing the effects of treating the endocrine factor or discontinuing the drug without a muscle biopsy in the toxin/endocrine group. DM can be diagnosed with typical skin presentations and skin pathology. ASS can be diagnosed with typical serology and co-existing interstitial lung disease.

## 4. Discussion

With the standardized protocol (Figure 1A), muscle biopsy yielded 85.5% (59 of 69) of definitive diagnosis, including 40.6% (28 of 69) changes in pre-biopsy diagnosis and 49.3% (34 of 69) narrowing down the suspicious myopathies. Among those with changes in diagnosis, 39.3% (11 of 28) had corresponding changes in treatment, which benefits the patients timely and significantly. In contrast, the definitive diagnosis of myopathy without a muscle biopsy was 59.3% (80/135), mainly achieved by serological tests, identifying co-existing diseases, and therapeutic trials.

For evaluating the effects of muscle biopsy, the nested one-group pretest-posttest quasi-experimental design suffered from the limitations of the absence of a control group and the non-random allocation. However, we have to compromise because the randomization can be unethical, while the muscle pathology was thought as a helpful tool in making a final diagnosis before coming to the high throughput molecular diagnosis era. The analysis for patients diagnosed with muscle disease without muscle biopsy in the same period shown in Result 3.4 was the alternative way to compensate for the possible bias of one group design.

In past decades, patients with myopathy had the relatively low intention of knowing their final diagnosis through an invasive procedure because of lacking effective treatment. However, with the refined classifications of myopathy, the advances in serology studies, staining markers, high throughput molecular diagnosis, and corresponding treatments, muscle pathology became necessary for precision diagnosis and treatment. With an optimized protocol, the diagnostic yields in adult myopathy can be raised from around 50% to 85%, which guides the subsequent precision treatments [19,21,47].

In the diagnostic workup, EMG and MRI provided an overall of 60–65% evidence for myopathic change. In comparison, muscle biopsy yielded an 85.5% definite diagnosis. Neither EMG nor MRI alone was enough to distinguish muscle disease categories and give a definitive diagnosis. Myopathic EMG changes can be masked by chronic neuropathic change due to underlying medical conditions. For example, neuropathic findings in EMG are unavoidable in a patient with long-lasting type 2 diabetes. Thus, muscle biopsy provided information for making a final diagnosis in patients with typical myopathy presentations but lack of myopathic change in EMG (Table 5).

Regarding the muscle MRI, although a specific myopathic pattern may present in muscle MRI of IIM [31], it is not always helpful in subgrouping IIMs. Furthermore, fatty infiltration and/or edematous change does not necessarily indicate a pure myopathic process in muscle MRI [48]. Serology test for IIM increased the subtype diagnosis accuracy and, on the other hand, it sometimes misled the diagnosis because of the imperfect sensitivity and specificity (Table 2).

The recent advances in high throughput molecular diagnoses, which help confirm hereditary muscle disorders, impact the priority of muscle biopsy. However, hereditary myopathy is relatively rare in adults. Muscle pathology still guides the subsequent choice of adequate laboratory tools. For instance, a female whose pre-biopsy diagnosis was inflammatory myopathy was confirmed to be a lipid storage myopathy by pathology. The subsequent tandem mass spectrometry and Sanger sequencing for the ETFDH gene confirmed the diagnosis of MADD. Another female whose initial diagnosis was autosomal recessive congenital myopathy had muscular dystrophy on muscle pathology. Due to asymmetric atrophy of thigh muscles on MRI, a Southern blot was applied to confirm the diagnosis of facioscapulohumeral muscular dystrophy (FSHD). The high throughput WES method may not be used in patients whose initial diagnosis was myositis and may not diagnose FSHD. In addition, in our cohort, a normal muscle pathology confirmed the pseudo-deficiency of acid α-glucosidase (GAA) activity in a subject carrying homozygous c.1726G > A (p.Gly576Ser) and c.2065G > A (p.Glu689Lys) [49,50]. (Table 5)

In adult-onset myopathy, inflammatory/autoimmune myopathies were the most common diagnosis, whether before or after muscle biopsy. Among patients with pre-biopsy diagnosis as IIM, 53% finally did not remain IIM, and 41% had changes in their diagnosis of IIM subtypes. Even without modification of the main category of myopathy, the changes in the subtype of IIM are of clinical significance. Subtypes of IIM are highly relevant to the effectiveness of treatments, the frequency of concomitant ILD, and the ratio of concurrent malignancy [51,52]. The accurate diagnosis of IIM subtypes guides clinicians on the frequency and extensiveness of cancer surveillance [53,54] and suggests the prognosis of pulmonary complications [37,51,52,55,56,57,58]. In addition, unlike most primary muscle diseases, IIMs are treatable and should not be misdiagnosed as other hereditary or degenerative myopathies. In this study, 6.9% of patients with other myopathies were finally proved to be IIM and subsequently underwent immunotherapy. Moreover, biopsy helped to identify steroid-induced myopathy in patients with IIM. Deterioration of muscle strength in IIM can be the fluctuated nature of the disease or a complication of long-lasting use of steroids. The two conditions are sometimes undistinguished but commonly encountered in clinics, while other systemic manifestations are occult. In this study, two patients underwent muscle biopsy for this reason, and the findings on histopathology gave a clear guide for the following treatment.

Special issues that alter the weight of muscle biopsy in reaching the final subtype diagnosis of IIM were (1) the sensitivity and specificity of serology tests and (2) the presence of typical skin manifestations of DM. In our cohort, 39 patients with IIM did not undergo a muscle biopsy, 44 underwent muscle biopsy, and 18 had a diagnosis change, including the shift of IIM subtype. In patients with typical skin features of DM and typical myositis-specific antibodies in serology, physicians may be more confident in making a final diagnosis without muscle biopsy, or the definitive diagnosis may be reached by skin biopsy in DM.

Metabolic myopathy is another category of muscle disease that is possibly treatable. The presentations of elevated CK, myalgia, weakness, and even rhabdomyolysis made it sometimes indistinguishable from IIM. Two of 18 IIMs (11.1%) were diagnosed as metabolic myopathy after biopsy. One is MADD, which can be effectively treated with carnitine supplements. Although the case number of metabolic myopathy is small, it can be myositis mimicking and is undoubtedly worth finding out by muscle biopsy.

Congenital or hereditary myopathies were of low prevalence and rarely diagnosed in adult patients in this study, making it hard to weigh the molecular diagnosis and muscle biopsy. Genetic analyses were introduced to a few cases whose muscle pathology indicated dystrophy even though no apparent family history was identified. Although muscle biopsy provides a precise guide for choosing the subsequent molecular tool in adult patients, early introduction of genetic tests before muscle biopsy may benefit pediatric patients regarding the risk of general anesthesia for biopsy [59,60].

According to the results of our pretest-posttest study, muscle biopsy changed the pre-biopsy diagnosis in 40% of patients. Therefore, in our post hoc analysis for myopathy patients without muscle biopsy, those diagnosed by serology tests or therapeutic trials may still be at the risk of misdiagnosis. In addition, for the 55 patients who had an uncertain type of myopathy or inconclusive results from electrophysiological tests, muscle biopsy might help. The hesitancies for muscle biopsy were from both patients and clinicians. The major hesitancies from patients included the safety of the invasive procedure and insufficient insurance coverage. The concerns from clinicians included the confidence of making a final diagnosis, the lack of subsequent treatment after the definite diagnosis was made, and unexpected surgical complications, especially in patients with co-morbidities. This cohort had no biopsy-related surgical complications, consistent with previous reports [60,61,62,63]. This data can provide for patients considering this procedure in the future.

Our protocols for muscle MRI, muscle biopsy, specimen transportation, EMG for myopathy were optimized, standardized, and operated well. The standardized report forms for muscle MRI, muscle pathology, and EMG had been applied for years. The inter-rater bias of histopathology was minimized [64]. However, the retrospective nature of this study suffered from several challenges such as (1) it was not able to recapitulate the considerations while the pre-biopsy diagnosis was made entirely; (2) The use of anti-inflammatory agents before muscle biopsy, which may obscure pathological interpretation, was not able to be avoided (51 out of 69 patients had received medical treatment before biopsy); and (3) there was no chance to reduce the relatively higher ratio of missing data: missing EMG in 9 patients (13%) and MRI in 9 patients (13%).

To sum up, our results suggested that with a careful, completed protocol, muscle biopsy provides high diagnostic value in precision diagnosis for clinically indistinguishable adult-onset myopathies [17,18,21,23,59,63,65,66,67]. This study analyzed the change of the latest IIMs subtypes before and after muscle biopsy. With more precise diagnoses of IIMs subgroups being made after a muscle biopsy, a more proper treatment following the accurate diagnosis will be given, which will provide a better quality of patient care.

## Figures and Tables

**Table 1:** Demographic and clinical information of patients with muscle biopsy.

	Total (*n* = 69)	Without Change (*n* = 40)	With Change (*n* = 29)	*p*-Value
Age (year, mean ± SD)	54 ± 13.7	55 ± 13.8	53 ± 13.9	0.258
Gender (male, *n* (%))	27 (39.1)	18 (45)	9 (31)	0.126
CK level (U/L, mean ± SD)	3031 ± 7409	2468 ± 4239	3703 ± 10,282	0.495
EMG findings (*n* (%))				0.517
Myopathic change	43 (62.3)	25 (62.5)	18 (62.1)	
Neuropathic change	6 (8.7)	2 (5)	4 (13.8)	
Mixed	9 (13)	6 (15)	3 (10.3)	
Normal	2 (3)	2 (5)	0 (0)	
Not available	9 (13)	5 (12.5)	4 (13.8)	
Muscle MRI (*n* (%))				0.909
Abnormal findings	46 (66.7)	25 (62.5)	21 (72.4)	
Unremarkable	14 (20.3)	11 (27.5)	3 (10.3)	
Not available	9 (13)	4 (10)	5 (17.3)	
Pre-biopsy primary diagnosis (*n* (%))				1.000
Inflammatory/Autoimmune	44 (63.8)	26 (65)	18 (62)	
Toxin/Endocrine	2 (2.9)	0 (0)	2 (7)	
Metabolic/Mitochondrial diseases	8 (11.6)	5 (12.5)	3 (10)	
Others	15 (21.7)	9 (22.5)	6 (21)	
Pre-biopsy treatment (*n* (%))				0.990
Steroid/immune-modulating therapy	38 (55.1)	24 (60)	14 (48.3)	
Discontinue myotoxic agent	3 (4.3)	0 (0)	3 (10.3)	
Other types of medication	10 (14.5)	5 (12.5)	5 (17.3)	
No treatment	18 (26.1)	11 (27.5)	7 (24.1)	

SD, standard deviation; CK, creatine kinase; EMG, electromyography; MRI, magnetic resonance imaging.

**Table 2:** A list of patients who were misdiagnosed as IIM before muscle biopsy.

Case	Post-Biopsy Diagnosis	Clinical Information	
1	Metabolic myopathy (MADD)	Clinical presentation	A 37-year-old female diagnosed with polymyositis for years without muscle biopsy had yearly deterioration. She had a poor response to immune therapy, even with rituximab. The steroid was discontinued for an extended period.
		NE	Axial weakness and proximal limb weakness with MRC scale 3 over 4 limbs.
		Lab	CK level (505 U/L); positive anti-PM-Scl 75 antibody; negative acetylcholine receptor antibody The MS/MS for various lengths of fatty acid was done after a muscle biopsy, which showed elevations of long- to mid-chain fatty acid.
		NCS/EMG	Essentially normal.
		MRI	Diffuse muscular swelling and enhancement at bilateral thighs, especially at soleus muscles.
		Muscle pathology	Myopathic changes with intracellular lipid accumulation (Sudan III and oil-red-O are both positive). Type 1 muscle atrophy with focal type 2 muscle grouping without active inflammatory myopathy. The result suggested metabolic myopathy.
		Diagnosis and outcome	The genetic test confirmed the compound heterozygous mutation of the ETFDH gene. The patient completely recovered after the carnitine supplement.
2	Muscular dystrophy	Clinical presentation	A 45-year-old female with insidious onset, progressive bilateral lower limbs weakness for 3 years
		NE	Proximal weakness with MRC scale 4 over bilateral lower limbs and positive Gowers’ sign.
		Lab	CK level (7354 U/L); elevated liver enzymes.
		NCS/ENG	Diffuse myopathic change with some neuropathic change.
		MRI	Diffuse muscular atrophy with fat replacement of bilateral thighs.
		Muscle pathology	Marked fiber degeneration and regeneration, along with occasional fiber necrosis and endomysial fibrosis. Despite the degenerative change, clumps or chain of nuclei were rarely seen. About 10% of fibers show internal nuclei. There was mild and focal endomysial infiltration of mononuclear cells, consist of mainly T-lymphocytes. No excessive storage of glycogen or intracellular lipid. The result suggested a muscular dystrophy.
		Diagnosis and outcome	Molecular diagnosis was not done, because the patient was lost to follow-up after being discharged.
3	Metabolic or mitochondrial myopathy	Clinical presentation	A 51-year-old male had polymyositis under daily prednisolone 30 mg. Subacute onset bilateral lower limbs weakness for 2 years.
		NE	Proximal weakness over 4r limbs; positive Gowers’ sign.
		Lab	Elevated CK level (1197 U/L); absence of myositis autoantibodies.
		NCS/ENG	Myopathic changes without irritability.
		MRI	Non-specific, minimal edema with asymmetric distribution at the muscles of both thighs.
		Muscle pathology	Minimal myopathic changes with presence of intracellular lipid deposition. Gomori trichrome showed increased mitochondria, COX staining was intact, but SDH staining was lost. The result suggested either metabolic or mitochondrial myopathy.
		Diagnosis and outcome	Molecular diagnosis was not done because the patient was lost to follow-up after being discharged.
4	Sarcopenia	Clinical presentation	A 72-year-old male ILD was in the treatment for pulmonary tuberculosis had insidious onset progressive exertional dyspnea and general weakness for four months.
		NE	Atrophy over the bilateral shoulder and pelvic girdles, proximal weakness with MRC scale 4, and positive Gowers’ sign.
		Lab	Normal CK level; presence of anti-Ku and anti-PL-12 antibodies.
		NCS/ENG	Neuropathic changes.
		MRI	Non-specific, minimal edema with symmetric distribution of both gluteal and thigh areas.
		Muscle pathology	Myopathic changes with predominant type 2 muscle atrophy, worst in type 2B muscles. Electronic microscopic findings showed degenerative changes and sarcolemmal fold in some atrophic fibers. Sarcopenia secondary to malnutrition was considered.
		Diagnosis and outcome	He showed much improvement after nutritional support and regained body weight 8 months later.
5	Mycobacterium-related granulomatous myopathy	Clinical presentation	A 61-year-old female had completed the modified radical mastectomy and combined chemo-radiotherapy for her breast cancer. She had subacute onset left hand and bilateral lower limbs progressive swelling and weakness for one month.
		NE	MRC scale 4 of bilateral upper limbs, MRC scale 5 of bilateral lower limbs, and preserved deep tendon reflexes.
		Lab	CK level (3364 U/L); elevated liver enzymes; positive ANA-cytoplasm; elevated rheumatoid factor.
		NCS/ENG	Sensorimotor polyneuropathy and irritable myopathy.
		MRI	Infiltration and edema at subcutaneous regions and muscles suggesting dermatomyositis.
		Muscle pathology	Granulomatous inflammation with necrosis and positive immunostain with CD 68. The acid fast stain was negative. Skin biopsy: palisading necrotizing granulomatous dermatitis. The causative pathogen was identified as Mycobacterium haemophilium.
		Diagnosis and outcome	After completing clarithromycin and ciprofloxacin treatments, she was recovered entirely many months later.
6	Fibromyalgia	Clinical presentation	A 67-year-old female had insidious onset neck tightness, neck weakness, and fatigue for years.
		NE	Full muscle strength.
		Lab	Normal CK level; weakly positivity for anti-SRP and anti- SAE1.
		NCS/ENG	Myopathic change.
		MRI	Cervical spine MRI showed herniated intervertebral discs at multiple levels.
		Muscle pathology	Mild myopathic change without specific pattern.
		Diagnosis and outcome	After excluding myopathy, duloxetine was tried, and the patient got extraordinary improvements.
7	Diabetic amyotrophy	Clinical presentation	A 68-year-old T2DM male with rosuvastatin use had acute onset lower limbs weakness and lower backache during hospitalization for infection of unknown origin.
		NE	MRC scale 2 and 4 on bilateral proximal and distal lower limbs, respectively.
		Lab	Normal CK level and absence of myositis autoantibodies.
		NCS/ENG	Bilateral upper lumbar radiculopathy.
		MRI	Spinal MRI showed posterior herniation of L4-5 disc with mild compression of thecal sac and nerve roots, and central herniations of C5-6 disc with compression of the thecal sac.
		Muscle pathology	Biopsy was performed due to suspicion of superimposed statin-induced myopathy. Diffuse atrophic fibers with minimal perivascular infiltration of mononuclear cells; almost absence of endomysial or perimysial infiltration of mononuclear cells.
		Diagnosis and outcome	The patient was diagnosed with diabetic amyotrophy. His muscle strength was improved 6 months later after strict glycemic control.
8	Steroid-related myopathy	Clinical presentation	A 66-year-old female with ILD, taking prednisolone, was admitted for pneumonia.
		NE	Muscle powers were full in 4 limbs, negative Gowers’ sign, presence of mechanic’s hand.
		Lab	Normal CK level; elevated anti-SSA antibody (184 U/mL); strong positive anti-Jo-1 and anti-Ro-52 antibodies.
		NCS/ENG	Sensorimotor polyneuropathy; myopathic changes in bilateral vastus medialis without irritability.
		MRI	Edematous change and rim enhancement at bilateral sartorius, gracilis and rectus femoris muscles.
		Muscle pathology	Chronic myopathic changes with type 2 fiber atrophy. No fiber necrosis or phagocytosis with minimal infiltration of mononuclear cells.
		Diagnosis and outcome	Azathioprine and pirfenidone were added for ILD. The steroid was not suspended because the benefit outweighs the adverse effect in her deteriorating clinical course.
9	Steroid-related myopathy	Clinical presentation	A 59-year-old female with hypothyroidism and skin disease treated with methylprednisolone. She presented with subacute onset progressive four limbs weakness for 3 months.
		NE	Drowsy consciousness, dysarthria, quadriparesis with MRC scale 2 on all limbs, areflexia except normal deep tendon reflexes on bilateral brachioradialis.
		Lab	Normal CK level with strong positivity for anti-PL-12 and anti-Ro-52 antibodies.
		NCS/ENG	NCS showed sensorimotor polyneuropathy. EMG was performed only at resting state because she could not cooperate for minimal and maximal effort due to decreased conscious level. Increased resting activities in right gastrocnemius, abductor pollicis brevis and semimembranosus muscles.
		MRI	Edema at the subcutaneous regions and muscles of bilateral gluteal regions and thighs, suggesting dermatomyositis. Septic arthritis of both hip joints, and minimal effusions of both knee joints.
		Muscle pathology	Severe type 2 fiber atrophy. No fiber necrosis, phagocytosis or presence of internal nuclei. Minimal endomysial infiltration of mononuclear cells and negative immunostain with MHC class I. The NADH-TR stain showed atrophic, degenerative fibers. COX, SDH and AMPDA were all intact. No excessive storage of glycogen or intracellular lipid. Steroid myopathy or hypothyroidism-related changes were suggested.

**Table 3:** A list of patients who had both diagnosis and treatment changed after muscle biopsy.

Case	Pre-Biopsy Main Diagnosis	Pre-Biopsy Differential Diagnosis	Post-Biopsy Diagnosis	Clinical Decision
1	SLE-related myopathy	CIDP or mononeuritis multiplex	Steroid-related myopathy	Steroid-sparing plaquenil as therapy
2	IIM (PM) with CTD and ILD	Pompe disease due to presence of paraspinal myotonia	Malnutrition sarcopenia	Nutritional support
3	IIM	Metabolic, endocrine, or drug-related myopathy	IIM (OM)	Began steroid therapy
4	Undetermined myopathy	Fibromyalgia	No evidence of myopathy	Began duloxetine therapy
5	Sjögren syndrome with IIM (DM)		IIM (OM)	Higher dose of steroid for therapy
6	IIM		Granulomatous myopathy due to Mycobacterium infection	Began treatment for Mycobacterium infection
7	Myasthenia gravis	Undetermined myopathy	Steroid-related myopathy	Steroid-sparing azathioprine therapy
8	IIM (PM)	Metabolic myopathy	Metabolic myopathy	Began carnitine-L therapy
9	Steroid-related myopathy	Worsening pre-existing IIM (PM)	Residual activity of IIM (PM)	Re-started steroid therapy
10	IIM (PM)		IIM (OM)	Higher dose of steroid
11	Metabolic myopathy	Congenital myopathy	Myopathy with positive Ro-52 myositis antibody	Began steroid and azathioprine therapy

SLE, systemic lupus erythematosus; IIM, idiopathic inflammatory myopathy; PM, polymyositis; DM, dermatomyositis; OM, overlapping myositis; CIDP, chronic inflammatory demyelinating polyneuropathy; CTD, connective tissue disease; ILD, interstitial lung disease.

**Table 4:** Change of pre- and post-biopsy diagnosis in patients with idiopathic inflammatory myopathies.

Diagnosis	Post-Biopsy Non-IIMs	Post-Biopsy IIMs	Total
Pre-biopsy IIMs	10	8	18
Pre-biopsy non-IIMs	7	4	11
Total	17	12	29

IIMs, idiopathic inflammatory myopathies; non-IIMs, non-idiopathic inflammatory myopathies.

**Table 5:** A list of patients undergone muscle biopsy whose EMG did not show myopathic change.

Case	Pre-Biopsy Diagnosis	Post-Biopsy Diagnosis	Clinical Information	
**EMG: Normal Findings**				
1	Inflammatory myopathy	Metabolic myopathy (MADD)	Clinical presentation	A 37-year-old female diagnosed with polymyositis had yearly deterioration for years without a muscle biopsy. She had a poor response to immune therapy, even with rituximab. The steroid was discontinued for an extended period.
			NE	Proximal weakness with MRC scale 3 over upper and lower limbs and truncal weakness.
			Lab	CK level 505 U/L, positive anti-PM-Scl 75 antibody, negative acetylcholine receptor antibody. The MS/MS for various lengths of fatty acid was done after a muscle biopsy, which showed elevations of long- and middle-chain fatty acid.
			MRI	Diffuse muscular swelling, infiltration, and enhancement at bilateral thighs, especially at soleus muscles.
			Muscle pathology	Myopathic changes with intracellular lipid accumulation (Sudan III and oil-red-O were positive). Type 1 muscle atrophy with focal type 2 muscle grouping without active inflammatory myopathy. The result suggested metabolic myopathy.
			Diagnosis and outcome	The genetic test confirmed the compound heterozygous mutation of the ETFDH gene. The patient completely recovered after the carnitine supplement.
2	Hereditary congenital myopathy	FSHD	Clinical presentation	A 61-year-old female had insidious onset, progressive proximal lower limbs and axial weakness for more than 5 years.
			NE	Diffuse muscle atrophy, MRC scale 4 over bilateral upper and lower limbs, negative percussion myotonia, diffuse hyperreflexia.
			Lab	Normal CK level.
			MRI	Muscle atrophy of left semitendinosus, semimembranosus, gastrocnemius muscles, bilateral vastus lateralis, intermedius, medialis muscles, and gluteus maximus muscles.
			Muscle pathology	Chronic myopathic changes with fiber necrosis, primarily type 1 fiber atrophy. No central core or excessive storage of glycogen by PAS stain. No rimmed vacuoles. No endomysial infiltration of mononuclear cells. No fatty spilling from the replacing adipose tissue.
			Diagnosis and outcome	The genetic test confirmed FSHD (D4Z4 deletion 27Kb). The patient needed a cane one year later.
**EMG: Neuropathic findings**				
3	Pompe disease	Pseudodeficiency of GAA	Clinical presentation	A 58-year-old male treated with a statin for his hyperlipidemia had insidious onset lower limbs predominant myalgia.
			NE	Preserved muscle strength and deep tendon reflex.
			Lab	Elevated CK level (730 U/L), reduced GAA activity, no abnormal organic acids found in the urine.
			MRI	Unremarkable.
			Muscle pathology	Normal appearing muscle; no rimmed vacuoles or ragged red fibers; no structural myopathy on NADH-TR stain. The COX, SDH and AMPDA are all intact. No excessive storage of glycogen or intracellular lipid.
			Diagnosis and outcome	Confirm psedodeficiency of GAA caused by c.1726G>A (p.Gly576Ser) homozygous and c.2065G>A(p.Glu689Lys) homozygous mutation. Statin was discontinued.
4	Congenital NMJ disorder or motor neuron disease	Motor neuron disease	Clinical presentation	A 25-year-old female had insidious onset, progressive dysphagia and general weakness for 3 months.
			NE	Atrophy over tongue, biceps, triceps, and deltoid; MRC scale 3 over right proximal upper limb and scale 4 on distal part, while the rest of muscle power was full; generalized hyperreflexia
			Lab	Normal CK level, more than 10% decremental change on RNST.
			MRI	No signal change at the muscles of both thighs.
			Muscle pathology	Minimal histological change with mild, variable fiber size and small angulated fibers. No endomysial fibrosis, fiber necrosis, phagocytosis, regeneration, internal nuclei, inclusions or infiltration of inflammatory cells. Ultrastructure images showed intact myofibers, regular Z lines, except for some small foci of mild loss of myofilaments.
			Diagnosis and outcome	WES showed FUS mutation. The patient underwent tracheotomy due to respiratory failure and expired after ventilator withdrawal in hospice care.
5	Non-specific myopathy	Non-specific myopathy	Clinical presentation	A 41-year-old female had insidious onset, progressive weakness over bilateral lower limbs and jaw, face muscle while chewing for one year.
			NE	Proximal weakness of MRC scale 4 over all limbs, and positive Gowers’ sign.
			Lab	Normal CK level, negative autoimmune serology tests, normal GAA activity; absence of abnormal organic acid in urine.
			MRI	No image evidence of muscular atrophy or edematous change.
			Muscle pathology	Scattered small, angulated fibers without grouping. No inflammatory cells indented. Unremarkable findings by Gomori trichome and NADH-TR stain. COX, SDH, and AMPDA were intact. No excessive storage of glycogen or intracellular lipid.
			Diagnosis and outcome	WES showed a negative result.
6	Inflammatory myopathy	Sarcopenia	Clinical presentation	A 72-year-old male had interstitial lung disease. He was in the treatment for pulmonary tuberculosis and had insidious onset progressive exertional dyspnea and general weakness for four months.
			NE	Atrophy over the bilateral shoulder and pelvic girdles, proximal weakness with MRC scale 4, and positive Gowers’ sign.
			Lab	Normal CK level, presence of anti-Ku and anti-PL-12 antibodies.
			MRI	Non-specific, minimal edema with symmetric distribution of both gluteal and thigh areas.
			Muscle pathology	Myopathic changes with predominant type 2 muscle atrophy, worst in type 2B muscles. Electronic microscope showed degenerative changes and sarcolemmal fold in some atrophic fibers. Sarcopenia secondary to malnutrition was considered.
			Diagnosis and outcome	Nutritional support was the mainstay of treatment, and he showed much improvement after he regained body weight 8 months later.
7	Statin related myositis	Diabetic amyotrophy	Clinical presentation	A 68-year-old T2DM male using rosuvastatin had acute onset lower limbs weakness and lower backache during hospitalization for infection of unknown origin.
			NE	MRC scale 2 and 4 on bilateral proximal and distal lower limbs, respectively.
			Lab	Normal CK level and absence of myositis autoantibodies.
			MRI	Spinal MRI showed posterior herniation of L4-5 disc with mild compression of thecal sac and nerve roots, and central herniations of C5-6 disc with compression of the thecal sac.
			Muscle pathology	Chronic neuropathic changes, diffuse atrophic fibers with minimal perivascular infiltration of mononuclear cells. Almost absent of endomysial or perimysial infiltration mononuclear cells.
			Diagnosis and outcome	The patient was diagnosed with diabetic amyotrophy. His regained muscle strength 6 months later after strict glycemic control.
8	Inflammatory myopathy	IMNM	Clinical presentation	A 43-year-old male with T2DM and statin use had acute onset bilateral thighs pain for one day; relapse and remitting a few times.
			NE	Positive Gowers’ sign, diffuse hyporeflexia
			Lab	Elevated CK level (4284 U/L), weak positivity for anti-MDA5 antibody.
			MRI	Not performed.
			Muscle pathology	Active myopathic damage with necrotic fibers and minimal inflammatory infiltration. Samples were stained with ATPase 9.4, 4.3 and 4.6 and showed type 2 fiber predominance (particular 2B fiber). No inclusions, rimmed vacuoles, split fibers or lobulated fibers. No excessive storage of glycogen or intracellular lipid. The Gomori trichrome was unremarkable. The result suggested IMNM or myotoxic myopathy.
			Diagnosis and outcome	Urine organic acid and MS/MS were normal. Statin was discontinued.

MADD, multiple acyl-CoA dehydrogenation deficiency; FSHD, facioscapulohumeral muscular dystrophy; NMJ, neuromuscular junction; IMNM, immune-mediated necrotizing myopathy; NE, neurological examination; T2DM, type 2 diabetes mellitus; MRC, Medical Research Council scale for muscle strength; CK, creatine kinase; NCS, nerve conduction study; EMG, electromyography; NE, neurological examination; MS/MS, mass spectrometry; SRP, signal recognition particle antibody; RNST, repetitive nerve stimulation test; ETFDH, electron transfer flavoprotein dehydrogenase; WES, whole exome sequencing; GAA, acid alpha-glucosidase; COX, Cytochrome *c* oxidase; SDH, succinate dehydrogenase; AMPDA, adenosine monophosphate deaminase; NADH-TR, nicotinamide adenine dinucleotide dehydrogenase-tetrazolium reductase.

## Data Availability

Data are available upon reasonable request. Yuan-Ting Sun ORCID 0000-0003-2899-113.
